# Multifunctional Lipid-Based Nanoparticles for Codelivery of Anticancer Drugs and siRNA for Treatment of Non-Small Cell Lung Cancer with Different Level of Resistance and EGFR Mutations

**DOI:** 10.3390/pharmaceutics13071063

**Published:** 2021-07-11

**Authors:** Joydeb Majumder, Tamara Minko

**Affiliations:** 1 Department of Pharmaceutics, Ernest Mario School of Pharmacy, Rutgers, The State University of New Jersey, Piscataway, NJ 08854, USA; j.majumder@rutgers.edu; 2 Rutgers Cancer Institute of New Jersey, New Brunswick, NJ 08903, USA; 3 Environmental and Occupational Health Science Institute, Piscataway, NJ 08854, USA

**Keywords:** EGFR mutated and gefitinib-resistant NSCLC, lipid nanoparticles, siRNA, LHRH, paclitaxel

## Abstract

Resistance to chemotherapy, enhanced proliferation, invasion, angiogenesis, and metastasis (RPIAM) represent major obstacles that limit the efficacy of cancer treatment especially in advanced stages of cancer. Overcoming or suppressing RPIAM can dramatically improve the treatment outcome. Non-small cell lung cancer (NSCLC) is frequently diagnosed in an advanced stage and often possesses intrinsic resistance to chemotherapy accompanied by the fast development of acquired resistance during the treatment. Oncogenic receptor tyrosine kinases (TKs), specifically epidermal growth factor (EGF) TKs, play an important role in the activation of MAPK/PI3K/Akt/STAT pathways, finally leading to the development of RPIAM. However, the suppression of EGF-TK by different drugs is limited by various defensive mechanisms and mutations. In order to effectively prevent the development of RPIAM in NSCLC, we formulated and tested a multicomponent and multifunctional cancer targeted delivery system containing Nanostructured Lipid Carriers (NLCs) as vehicles, luteinizing hormone release hormone (LHRH) as a cancer targeting moiety, EFG-TK inhibitor gefitinib and/or paclitaxel as anticancer drug(s), siRNA targeted to EGF receptor (EGFR) mRNA as a suppressor of EGF receptors, and an imaging agent (rhodamine) for the visualization of cancer cells. Experimental data obtained show that this complex delivery system possesses significantly enhanced anticancer activity that cannot be achieved by individual components applied separately.

## 1. Introduction

Lung cancer represents the most common cause of cancer deaths in both men and women worldwide [1]. Non-small cell lung carcinoma (NSCLC) is the most common type of lung cancer, accounting for about 84% of cases [2]. The majority of NSCLC patients are diagnosed in the advanced or metastatic stage of the disease, when treatment options are limited to surgery, chemotherapy, few targeted therapies, and immunotherapy [2,3,4,5]. Consequently, the development of novel effective and safe approaches to treat this disease is vitally important. However, the limited clinical efficiency, toxicity, and development of resistance represent three critical barriers limiting progress in the therapy of NSCLC. Despite an initial response, metastatic lung cancer almost always eventually acquires resistance against all types of therapies, leading to poor survival rate in patients [6,7,8,9]. 

Oncogenic receptor Tyrosine Kinase (TK) pathways, specifically Epidermal Growth Factor (EGF) pathways, have been explored as targets for therapy of NSCLC, and EGF receptor (EGFR) inhibitors are currently used as first-line therapy options for patients with advanced stages of the disease [10,11]. Although monoclonal antibodies targeted to EGFR (e.g., cetuximab) and small-molecule TK inhibitors (e.g., erlotinib, gefitinib) may yield positive responses, these agents target only one type of EGFR, which is effective only in a small fraction of patients (about 10%), and they elicit numerous resistance mechanisms (e.g., T790M mutation). Moreover, small molecules are effective only in NSCLC patients with specific EGFR mutations (mostly deletions in exon 19 and nucleotide substitutions in exon 21) [12,13]. Consequently, patients with expression of EGFR but without these mutations usually do not respond to such treatment. New irreversible inhibitors of wild-type and mutant EGFR family members have effective antitumor activity but demonstrate a high toxicity profile and generally do not prevent the development of drug resistance [11,14]. The efficacy of most of the small molecule anticancer drugs is limited due to their low water solubility, poor site-specific bioavailability, high adverse side effects, and targeting of particular genetic types of EGFR. Similarly, biomacromolecule drugs such as small interfering RNA (siRNA) and messenger RNA (mRNA) are often degraded in body fluids thereby limiting their stability and concentration at the target site [15,16]. Nanosized delivery systems offer unique opportunities to protect and guide therapeutics toward the target site [17,18,19,20,21,22,23,24,25]. In addition, a nano drug carrier provides the opportunity to improve the solubility of small molecule drugs and the stability of bio-macromolecular drugs [26,27,28]. Furthermore, such a system could overcome the mucus barrier and poor lung penetration associated with systematic delivery [29,30,31,32]. Moreover, nanocarrier-based systems offer a unique ability to release the therapeutics at the target sites in a controlled and sustained way compared with the burst release in systematic methods [33]. Therefore, research efforts have been focused in the past decade on developing nanotherapeutics with improved therapeutic efficacy [34,35,36]. Over the years, a wide range of nanoscale drug delivery systems were exploited for treating various respiratory diseases including lung cancer [37,38,39,40,41,42,43]. Several nanosized delivery systems have also been developed for increasing the efficacy of EGFR TK inhibitors and overcome the development of resistance in lung cancer [23,24,44,45].

In recent years, paclitaxel containing various nanotherapeutics were also developed for the treatment of lung cancer [21,46,47,48]. As a result of poor water solubility, paclitaxel usually was loaded with Cremophor EL (CrEL) as a solubilizer and other surfactants. The use of such excipients caused high toxicity and adverse side effects, thereby limiting its clinical application [49]. Developing multifunctional and targeted nanocarrier-based formulations of paclitaxel can increase the water solubility of the compound, limit the drug accumulation in healthy organs and cells, and avoid using such excipients, thereby minimizing these side effects. 

Over the decade, various surface markers such as folate, epidermal growth factor receptor (EGFR), luteinizing hormone-releasing hormone (LHRH), etc., that are known to express on cancer cells, were explored for developing counter marker functionalized drug carriers to recognize the targeted diseased cells [50,51]. The surfaces of the nanocarriers were designed with targeting ligands that can interact with tumor-specific receptors such as EGFR [52], folate receptor [53,54], and LHRH receptor [55,56], providing for a targeted delivery of anticancer medicines specifically to lung cancer cells, increasing their bioavailability and finally resulting in enhanced anticancer efficacy and limited adverse side effects upon healthy organs, tissues, and cells [21,22,57,58,59,60,61,62]. Furthermore, to improve the stability of the nanocarrier-based delivery systems, the surface of the nanocarriers can be modified by acetylation [63] and with poly(ethylene glycol), PEG [20,64], since PEGylation reduces its interaction with the serum proteins and protects from the immune system [65]. In addition, lipid-based systems are structurally similar to the surfactant lining of the lungs, and thus, lipid-based nanomaterials have better retention time and less toxicity in the lungs [40,66,67,68,69]. In addition, lipid-based carriers allow for a local delivery of therapeutics directly to the lungs, further limiting their concentration in the systemic circulation and side effects [21,22,23,40].

In the current work, we address the above-mentioned important problems by substantially enhancing the efficiency of therapy and reducing its adverse side effects. Here, we have designed stable multifunctional nanostructured lipid carriers (NLCs) comprised of a PEG-coated surface that was conjugated with an LHRH analog as a targeting moiety for the specific delivery of anticancer therapeutics to NSCLC. Such NLCs were used for the delivery of the anticancer drug paclitaxel (PTX), the EGFR TK inhibitor gefitinib (GEF), a cancer cell-targeting moiety (LHRH), an imaging agent (rhodamine), and siRNA targeted to mRNA encoding EGF receptors. Cancer-targeted theranostic multicomponent and multifunctional NLCs were designed to simultaneously solve several different but closely related tasks: (1) detect cancer cells by targeting them with the LHRH peptide and visualizing them with the help of an imaging agent containing fluorescent dye; (2) protect, increase water solubility, and enhance the cellular internalization of active components by PEGylated stabilized lipid-based carries (NLCs); (3) induce cell death by the anticancer drug (PTX); (4) decrease the expression of EGF receptors by EGFR-targeted siRNA; and (5) inhibit EGFR TK by GEF. The experimental data obtained show that this complex multifunctional system possesses significantly enhanced anticancer activity that cannot be achieved by individual components applied separately.

## 2. Materials and Methods

### 2.1. Materials

Paclitaxel (PTX), gefitinib (GEF), triethylamine, α-tocopherol, trilaurin, and all other reagent and solvents were purchased from commercial sources and used directly in all the experiments. Cholesterol (Ovine), DSPE-PEG-2000-PE (1,2-distearoyl-sn-glycero-3-phosphoethanolamine-*N*-[methoxy(polyethylene glycol)-2000] (ammonium salt)), DSPC (1,2-distearoyl-sn-glycero-3-phosphocholine), DSPE-PEG-NHS (2000), and DOTAP (1,2-dioleoyl-3-trimethylammonium-propane (chloride salt)) were obtained from Avanti Polar Lipids (Alabaster, AL, USA) and used without further purification. A modified synthetic analog of LHRH deca-peptide with amino acid sequence Gln-His-Trp-Ser-Tyr-DLys(D-Cys)-Leu-Arg-Pro was synthesized based on our design [55,56] by the American Peptide Company, Inc. (Sunnyvale, CA, USA). All the drugs and NLCs stock solutions were diluted to the appropriate concentrations with growth medium immediately before use in cell culture assays.

### 2.2. Cell Lines

Human non-small cell lung cancer (NSCLC) cells A549, H-1975, PC-9, and PC-9GR were obtained from the American Type Culture Collection (ATCC, Manassas, VA, USA). H-1975 and PC-9 cells were cultured in RPMI-1640 growth medium (Sigma, Aldrich, St. Louise, MO, USA) supplemented with 10% fetal bovine serum (Fisher Chemicals, Fairlawn, NJ, USA) and 1% penicillin–streptomycin (Sigma, Aldrich, St. Louise, MO, USA); PC-9GR cells were grown in complete RPMI-1640 media containing an additional 1 µM of gefitinib. A549 cells were cultured in F-12K media supplemented with 10% fetal bovine serum and 1% penicillin–streptomycin. All the cells were cultured according to the ATCC protocol in a humidified atmosphere of 5% CO_2_ and 95% air (*v/v*) at 37 °C. All experiments were performed on cells in the exponential growth phase.

### 2.3. Synthesis of DSPE-PEG-LHRH Peptide

First, 34.0 mg LHRH peptide (0.025 mmol) was taken in 3 mL of THF/methanol mixture (2:1) in a 10 mL RB flask. Next, 50 µL triethyl amine was added dropwise to the reaction mixture and stirred for 10 min to activate the amine group of the peptide. Next, 50.0 mg of DSPE-PEG-NHS (2000) (0.025 mmol) was dissolved in 2 mL of THF and added to the RB flask under stirring condition. The reaction was continued for 24 h at room temperature. Next, the solvent was evaporated under reduced pressure at room temperature, and the colloidal suspension was transferred to a 3.5 kD MWCO dialysis tube, which was run against 1 L PBS buffer (pH 7.4). The dialysis was run for 48 h, and the buffer was changed after 6, 12, 24, and 36 h. The final product was lyophilized to get the target DSPE-PEG-LHRH peptide as a solid powder.

### 2.4. Preparation of NLCs

All the NLC formulations were prepared by the standard ultrasonic dispersion method. Paclitaxel (PTX) or gefitinib (GEF) were directly added to the hot lipid phase containing 35 mg trilaurin (solid lipid), 15 mg α-tocopherol (liquid lipid), 8.5 mg DSPC (emulsifier), and 10 mg DSPE-PEG-2000 (surfactant). The aqueous phase contained 5 mM NaCl, 5 mM TES, and 10% ethanol (pH 7.2). Then, 2 mL of aqueous solution was added slowly to the hot lipid phase at 80 °C and dispersed using a high-speed homogenizer for 30 min to get each of the NLC formulations. After the sonication, the hot emulsion was further diluted with 8 mL of the ice-cold aqueous phase, and the mixture was stirred for 1 h at room temperature. To prepare LHRH-coated NLC, 5 mg DSPE–PEG–LHRH peptide was added to the lipid phase. All the NLC formulations were stored at 4 °C for further use.

### 2.5. Preparation of NLC–siRNA Complexes

To prepare NLC–siRNA, the following procedure was performed. Five mg DOTAP (cationic lipid) was added in the lipid phase. The NLC–siRNA complexes were prepared according to our previously published protocol by adding anionic siRNA solution into the prepared DOTAP containing cationic NLCs [23]. siGENOME, the pool of siRNAs targeted to EGF receptors (containing four EGFR-specific siRNA duplexes) was obtained from Dharmacon (Lafayette, CO, USA). The sample mixtures were stirred at room temperature for 1 h to ensure a formation of the NLC–siRNA complex.

### 2.6. Preparation of Rhodamine Labeled NLCs

One mg of commercially available rhodamine-conjugated lipid (Thermo Fisher Scientific, Branchburg NJ, USA) was added into the lipid phase during the preparation of the NLC–GEF and LHRH–NLC–PTX–siRNA formulations.

### 2.7. Particle Size, Polydispersity Index, and Zeta Potential

The particle size distribution, polydispersity index (PDI), and the zeta potential of all the NLC formulations were measured by Malvern Zetasizer Nanoseries (Malvern PANalytical, Westborough, MA, USA) at room temperature. Each of these parameters was measured three times, and results were expressed as average value with SD.

### 2.8. Stability of NLCs under Storage Conditions

All formulations of NLCs were stored for 60 days at 4 °C in both aqueous phase and RPMI-1640 growth media. The stability of these NLCs was checked by measuring particle size, PDI, and zeta potential at various time periods. All NLCs formulations were also monitored by visual inspection to detect any precipitation or gelling process. All the measurements were performed in triplicate, and results were expressed as average value with SD.

### 2.9. Stability of NLCs under Low pH Conditions

All NLC formulations were tested in phosphate buffer saline with pH 4.5. One mL of each NLC was added to 100 mL PBS with pH 4.5, and the mixture was incubated for 3 h at 37 °C. Each sample mixture was tested for monitoring particle size and PDI after 0 and 3 h of incubation. All the measurements were performed in triplicate, and the results were expressed as average value with SD.

### 2.10. Stability of NLCs under Freeze/Thaw Conditions

The stability of all NLC formulations was checked under accelerated freeze/thaw conditions. All NLC formulations were subjected to three freeze–thaw cycles (one freeze cycle represents 12 h storage at −20 °C and one thaw cycle represents 12 h storage at room temperature) followed by sonication and being centrifuged for 5 min. After that, all NLCs were tested for monitoring particle size, zeta potential, and PDI measurements.

### 2.11. Drug Entrapment Efficiency (EE) and Drug Loading (DL) 

The percentages of drug entrapment efficiency (EE) and drug loading (DL) capacity into the NLCs were determined by the filtration/centrifugation method. Briefly, 1.0 mL of NLC suspension was centrifuged for 10 min at 10,000 rpm in a 10 kD MWCO dialysis tube. The amount of unentrapped paclitaxel in the filtrate receiver was analyzed by high-performance liquid chromatography (HPLC) system equipped with a UV spectroscope at 225 nm. The EE (%) was determined by calculating the ratio of drug entrapped in the NLCs to the initial amount of drug added, while the DL (%) was determined by calculating the ratio of drug entrapped in the NLCs to the total amount of lipids added.

### 2.12. HPLC Method

Waters binary high-performance liquid chromatography—HPLC (Waters Corporation, Milford, MA) was used for the measurement of drug entrapment efficiency and drug-loading capacity into the NLCs. Mobile phase: 60% acetonitrile and 40% water; flow rate: 1.0 mL/minute; injection volume: 10 µL, temperature: 25 °C, wavelength: 225 nm for gefitinib and 254 nm for paclitaxel, column: C18. The retention time of standard samples of gefitinib and paclitaxel was 3.13 and 6.67 min, respectively.

### 2.13. siRNA Conjugation Efficiency

The conjugation efficiency of human EGFR siRNA into the NLCS was determined by the standard SYBR Gold assay. Briefly, 50 µL of siRNA containing NLC samples was added into the black Nunc 96-well plate followed by the addition of 25 µL of the dilution buffer (nuclease free water). Next, 25 µL of 4× SYBR Gold solution was added, and the obtained mixture was incubated at room temperature in the dark for 15 min. Fluorescence intensity was measured at an excitation wavelength of 485 nm and an emission wavelength of 520 nm.

### 2.14. In Vitro Drug Release Studies

The in vitro release of drugs from the NLCs was studied using the dialysis bag method. First, 1 mL of each of the NLC–GEF and LHRH–NLC–PTX–siRNA was loaded into the dialysis bag with a 10 kD MWCO (Spectra/Pro Float-A-Lyzer G2 Dialysis Device), and then, it was placed in a 50 mL Falcon conical tube containing 25 mL of dialysis buffer (PBS, pH 7.4, 1% *v/v* Tween-20). Then, the conical tubes were placed on magnetic stir at room temperature. Then, 1 mL of each sample was withdrawn from the receiver solution, and each withdrawn sample was replaced with 1 mL of fresh dialysis buffer at various time points. Finally, the samples were analyzed for the drug content by the HPLC method.

### 2.15. Cytotoxicity Assay

The cytotoxicity of all NLC formulations was assessed by a standard 3-(4,5-dimethylthiazol-2-yl)-2,5-diphenyltetrazolium bromide (MTT) cell proliferation assay in A549, H-1975, PC-9, and PC-9GR human lung cancer cells. Briefly, 5000 cells in 100 μL of media were seeded into each well of 96-well plates and incubated 24 h at 37 °C at 5% CO_2_ and 95% air atmosphere. Then, media were aspirated and substituted with fresh media containing studied formulations. After 72 h of incubation, the old media in each well was replaced with 100 µL of the MTT dye solution (1 mg/mL), and the plate was incubated at 37 °C for 3 h. Next, 100 μL DMSO was added to each well to dissolve the formazan produced by mitochondrial reductase from live cells. Finally, the plate was put on an orbital shaker for 15 min at room temperature, and the absorbance of formazan was measured at 570 nm using a microplate reader. Cell viability was calculated as the percentage of cells remaining viable in reference to the untreated vehicle cells. The 50% inhibitory concentration (IC_50_) values were calculated using GraphPad Prism 7 software (GraphPad Software, La Jolla, CA, USA). All experiments were done with three technical replicates and were repeated three times for biological variation.

### 2.16. Cell Imaging

Five thousand cells were seeded in each well of a glass-bottom chamber and grown for 24 h. Then, cells were treated with rhodamine (red fluorescence) labeled NLCs for 24 h followed by staining with DAPI (blue fluorescence) for the nucleus. Finally, cells were washed three times with PBS to remove any trace of staining reagent, and images were captured using a 20× object in a fluorescent microscope. The nucleus is shown in blue and the presence of a fluorophore tag NLC is shown in red.

### 2.17. Western Blot

Approximately 1 × 10^6^ cells were seeded in each well of a 6-well plate. The next day, cells were treated with 1 µM of drugs/NLCs or fresh media as vehicle control, and the treatment was continued for 24 h in a humidified incubator at 37 °C and 5% CO_2_ atmosphere. Following 24 h treatment, the culture media were removed, and cells were washed with PBS. To obtain cellular lysates, 0.6 mL of ice cold RIPA buffer (supplemented with 1× protease inhibitor cocktail tablets) was added to each well in a 6-well plate, and the plate was gently rocked for 15 min on ice. Then, cells were transferred to microcentrifuge tubes and sonicated in ice-cold water for 1 min and incubated on ice for 45 min. Next, cell lysate was centrifuged at 10,000× *g* for 15 min at 4 °C. The supernatant fluid (total cell lysate) was transferred to a new microcentrifuge tube. The protein concentration of the total cell lysate was quantified by Pierce BCA Protein Assay (Thermo Fisher Scientific, Branchburg NJ, USA). Then, the cell lysate (60 µg of lysate protein per lane) was run through electrophoresis using 4–12% SDS-PAGE gel at constant 200 volt for 60 min. Proteins were transferred from the gel to PVDF transfer membrane using an electroblotting apparatus at constant 100 mA for 90 min. The nonspecific binding was blocked by incubating the membrane in 5% non-fat milk made in PBS-T (0.05% Tween-20 in PBS buffer) for 1 h. Then, the membrane was incubated with anti-EGFR primary antibodies overnight at 4 °C, and GAPDH was used as a loading control. Next, the membrane was washed three times (10 min each time) with TBS-T buffer and incubated with horseradish peroxidase-conjugated IgG secondary antibody for 2 h. Finally, the membrane was washed three times (10 min each time) with TBS-T buffer, and the protein bands were developed using a chemiluminescent reagent (Thermo Fisher Scientific, Branchburg NJ, USA) and visualized in BIO-RAD ChemiDoc Imaging System (BIO-RAD Laboratories, Hercules, CA, USA).

### 2.18. Statistical Analysis

Data were analyzed using descriptive statistics, single-factor analysis of variance (ANOVA), and presented as mean values ± standard deviation (SD) from four to ten independent measurements. The difference between variants was considered significant if *p* < 0.05.

## 3. Results

### 3.1. Design of Multifunctional NLCs

**Figure 1:**
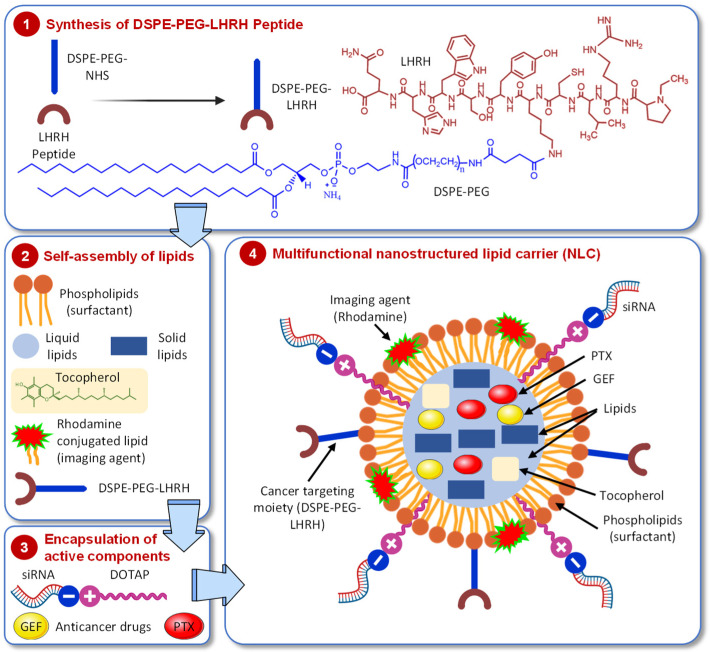
Schematic representation of cancer cell targeted multifunctional and multicomponent nanostructured lipid carrier (NLC). The scheme shows the design and four stages of preparation of the delivery system containing anticancer drugs (gefitinib, GEF, and/or paclitaxel, PTX), imaging agent (rhodamine), targeting moiety (DSPE-PEG-LHRH), and conjugated siRNA targeted to EGF receptors.

Nanocarriers possess unique properties such as small size, large surface area to volume ratio, etc. which allow them to carry various drugs, therapeutics, and imaging agents with high loading efficiency [70]. The earlier generations of monofunctional nanocarriers were capable of delivering a single therapeutic agent to overcome the shortcomings of its bioavailability, stability, and control release. The latest more complex nanocarrier systems called “multifunctional nanocarriers” were formulated to possess additional functions [71]. Such multifunctional nanocarrier systems can simultaneously perform many functions including delivery of therapeutics, disease-specific targeting, optical imaging, etc. Typically, multifunctional nanocarriers were designed by surface modification of the parent nanocarriers via covalent or non-covalent conjugation of affinity ligands selective for certain receptors on the target cell, cell-penetrating agents, imaging agents, stimuli-sensitive components, etc. In our current design of multifunctional lipid nanocarriers, we loaded the core structure of the NLC with the anticancer drugs gefitinib (GEF) and/or paclitaxel (PTX) and decorated the surface of the NLC with the LHRH targeting “DSPE–PEG–LHRH peptide”, which was synthesized by coupling DSPE–PEG–NHS with an amine functional peptide analog of LHRH (Figure 1, Step 1; Supplementary Materials Figure S1). Then, the self-assembly of lipids was performed by adding pheophorbides (surfactants) as well as liquid and solid lipids; rhodamine (imaging agent) was also conjugated (Figure 1, Step 2). Furthermore, a cationic lipid (DOTAP) was added to the membrane of NLC for non-covalent attachment of the human EGFR siRNA (siGENOME) in order to silence the EGFR gene in lung cancer cells (Figure 1, Step 3). 

**Figure 2:**
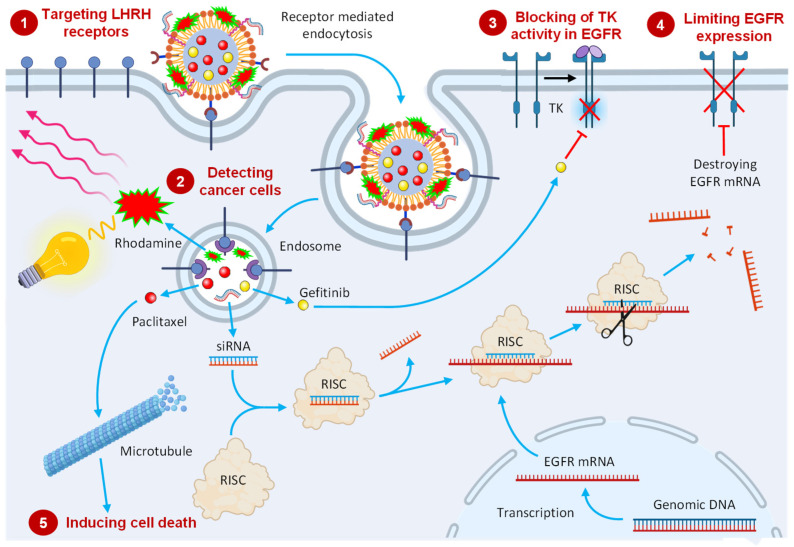
Schematic representation of internalization of a multifunctional cancer-targeted NLC-based delivery system with expected mechanisms of its anticancer action.

We hypothesize that the following mechanisms will be involved in the anticancer action of such a complex multifunctional delivery system (Figure 2). (1) Targeting of the system to LHRH receptors overexpressed in lung cancer cells initiates receptor-mediated endocytosis, destruction of the system in endosome/lysosome complex, and the release of active components of the system. (2) The combination of cancer targeting and imaging agent allows for the detection of tumor and spreading cancer cells by optical imaging. (3) Blocking EGF tyrosine kinase activity in existing EGFR by delivered GEF prevents the activation of the MAPK/PI3K/Akt/STAT pathway, limiting the proliferation, invasion, angiogenesis, and metastasis. (4) Delivered siRNA is loaded into the effector complex RNA-induced silencing complex (RISC), unwound during RISC assembly, and the single-stranded RNA hybridizes with target EGFR mRNA, leading to nucleolytic degradation of the targeted mRNA, decreasing the number of EGF receptors. (5) Escaped paclitaxel (PTX) affects microtubules in cancer cells, leading to the cell death. We expect that the complex interplay of these mechanisms will enhance the anticancer efficacy of active components to a degree that cannot be achieved by each of the component delivered separately. The following experiments were carried out in order to verify this hypothesis.

### 3.2. Preparation and Characterization of NLC Formulations

**Figure 3:**
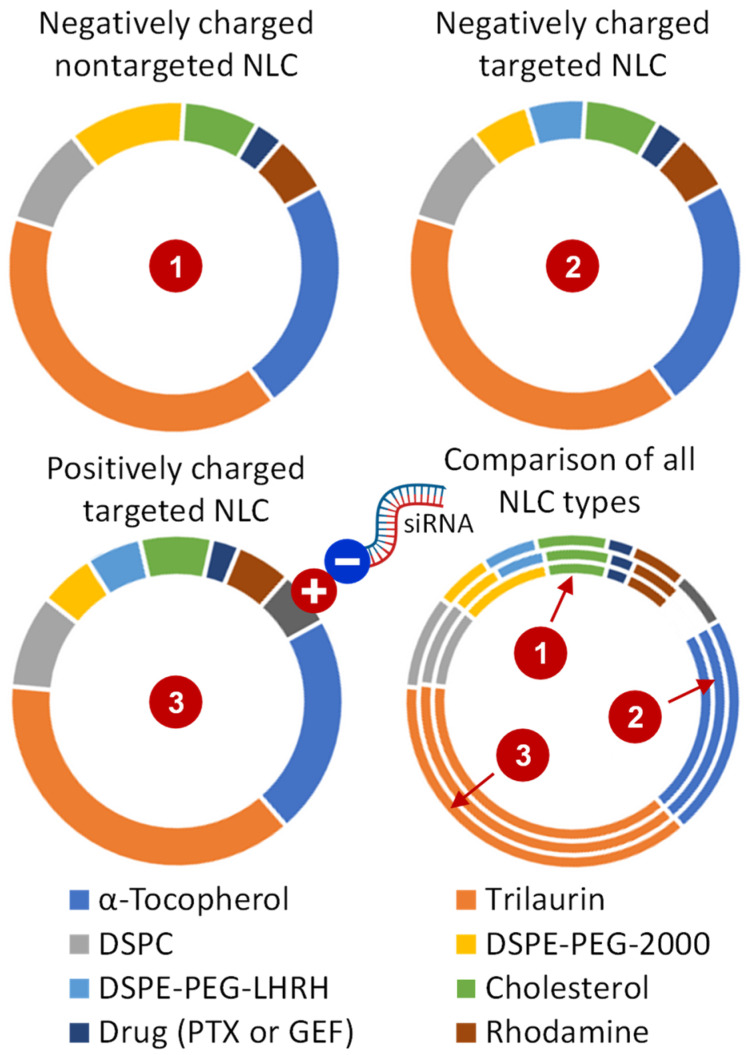
Compositions of NLC formulations.

All the NLC formulations were prepared by the probe sonication of the lipid phase in an aqueous phase following the standard ultrasonic dispersion method. PTX or GEF were directly added to the hot lipid phase containing solid lipid trilaurin, liquid lipid α-tocopherol, emulsifier DSPC (1,2-distearoyl-sn-glycero-3-phosphocholine), and surfactant DSPE-PEG-2000-PE (1,2-distearoyl-sn-glycero-3-phosphoethanolamine-*N*-[methoxy(polyethylene glycol)-2000] (ammonium salt)). The aqueous phase contained 5 mM NaCl, 5 mM TES, and 10% ethanol (pH 7.2). Then, 2 mL of aqueous solution was added slowly to hot lipid phase at 80 °C and dispersed using a high-speed homogenizer for 30 min to get each of the NLC formulations. After sonication, the hot emulsion was further diluted with 8 mL of the ice-cold aqueous phase, and the mixture was stirred for 1 h at room temperature. To prepare LHRH-coated NLCs, DSPE–PEG–LHRH peptide was added to the lipid phase. To attach the negatively charged siRNA on the surface of NLC, cationic lipid DOTAP (1,2-dioleoyl-3-trimethylammonium-propane (chloride salt)) was added in the lipid phase, and the mixtures of NLC and siRNA were stirred at room temperature for 1 h to ensure NLC–siRNA complex formation following our previously published protocol [21,22]. A list of all the tested NLCs is shown in Table 1, and compositions of all the NLC formulations are summarized in Figure 3.

**Figure 4:**
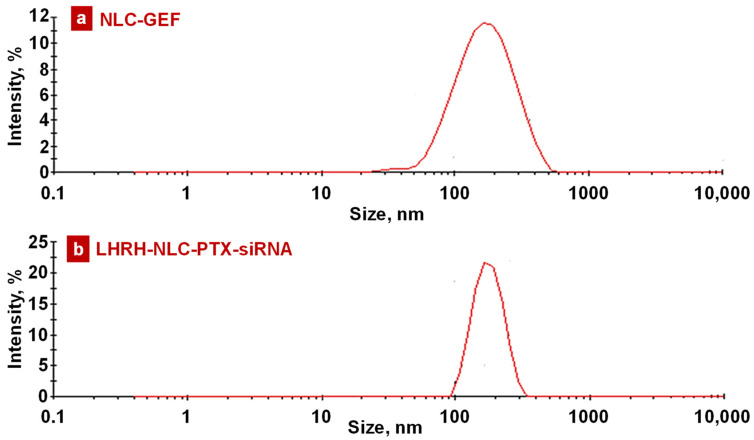
Representative histograms of intensity-size distribution of (**a**) NLC–GEF and (**b**) LHRH–NLC–PTX–siRNA.

All the NLCs were evaluated for particle size, polydispersity index (PDI), and zeta potential under various storage conditions. All the siRNA-conjugated NLCs displayed relatively small residual positive zeta potential, while NLC formulations without siRNA were slightly negative. Figure 4 and Figure S2 represent the size distribution of these NLCs. All the NLCs displayed a narrow size range with a single peak indicating a nearly monodispersed formulation. However, the size of some loaded nanoparticles varies substantially. Nevertheless, the distribution of nanoparticle size for the most important products (containing paclitaxel, siRNA, and LHRH peptide) was relatively sharp and monodispersed (Figure 4). Only less than 2–3% of these nanoparticles were smaller than 100 nm or larger than 300 nm. Previously, we found that such a range of sizes is the most effective for cancer-targeted nanoparticles and provided the most effective delivery and retention of anticancer drugs and nucleic acid in the lungs after inhalation delivery [72].

### 3.3. Stability of NLC Formulations

**Figure 5:**
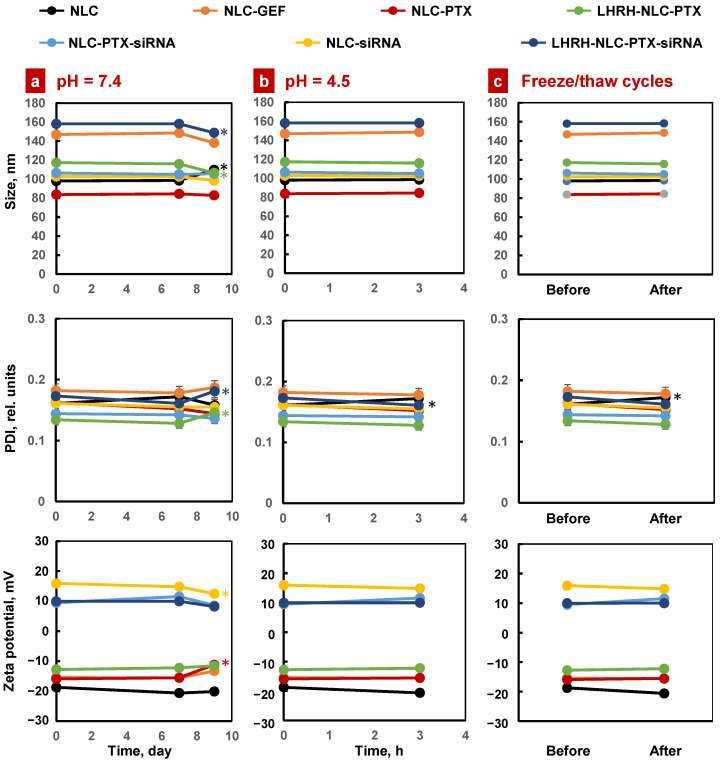
Stability of different NLC formulations in various storage conditions: (**a**) Aqueous solution with normal pH; (**b**) Aqueous solution with low pH; (**c**) Three freeze–thaw cycles (one freeze cycle represents 12 h storage at −20 °C and one thaw cycle represents 12 h storage at room temperature). Means ± SD are shown (note that in most cases, SD values are too small to be seen on the figure). * *p* < 0.05 when compared with the day 0.

The stability of all the NLC formulations was monitored over time under various conditions by recording particle size, polydispersity index (PDI), and zeta potential. All these NLCs were stored at 4 °C for a period of 60 days in aqueous buffer of pH 7.4 and growth media. No significant changes were observed in particle size, PDI, and physical appearance after 60 days of storage (Figure 5, Table S1). The stability of the NLC formulations was assessed in phosphate buffer saline of pH 4.5, as medium mimicking the average gastric pH value of infant following a standard procedure [73,74]. All these NLC formulations were found stable under low pH 4.5 condition for 3 h with almost no changes in particle size, PDI, and the physical appearance (Figure 5). Next, we checked the stability of all the NLCs under accelerated conditions. All the NLCs were subjected to three freeze–thaw cycles (one freeze cycle represents 12 h storage at −20 °C and one thaw cycle represents 12 h storage at room temperature). After this acceleration, most of the NLC formulations were found to be stable with almost no changes in particle size, PDI, and zeta potential (Figure 5).

### 3.4. Drug Entrapment Efficiency, Loading Capacity, and Drug Release

The percentages of gefitinib and paclitaxel entrapment efficiency and loading capacity of the NLCs were determined by the filtration/centrifugation method [75]. The amount of unentrapped paclitaxel in the filtrate receiver was analyzed by a high-performance liquid chromatography (HPLC) system equipped with a UV spectroscope at 225 nm for gefitinib and 254 nm for paclitaxel. The HPLC retention time of gefitinib (GEF) and paclitaxel (PTX) in standard sample and in the corresponding NLC formulations are shown in Figure S3. As presented below in Equations (1) and (2), the EE (%) and DL (%) were determined by calculating the ratio entrapped in the NLCs to the initial amount of drug added (for EE) or lipid added (for DL).

EE (%) = (Drug entrapment in the NLCs/Drug added) × 100%(1)

DL (%) = (Drug entrapment in the NLCs/Total amount of lipids added) × 100%(2)

**Figure 6:**
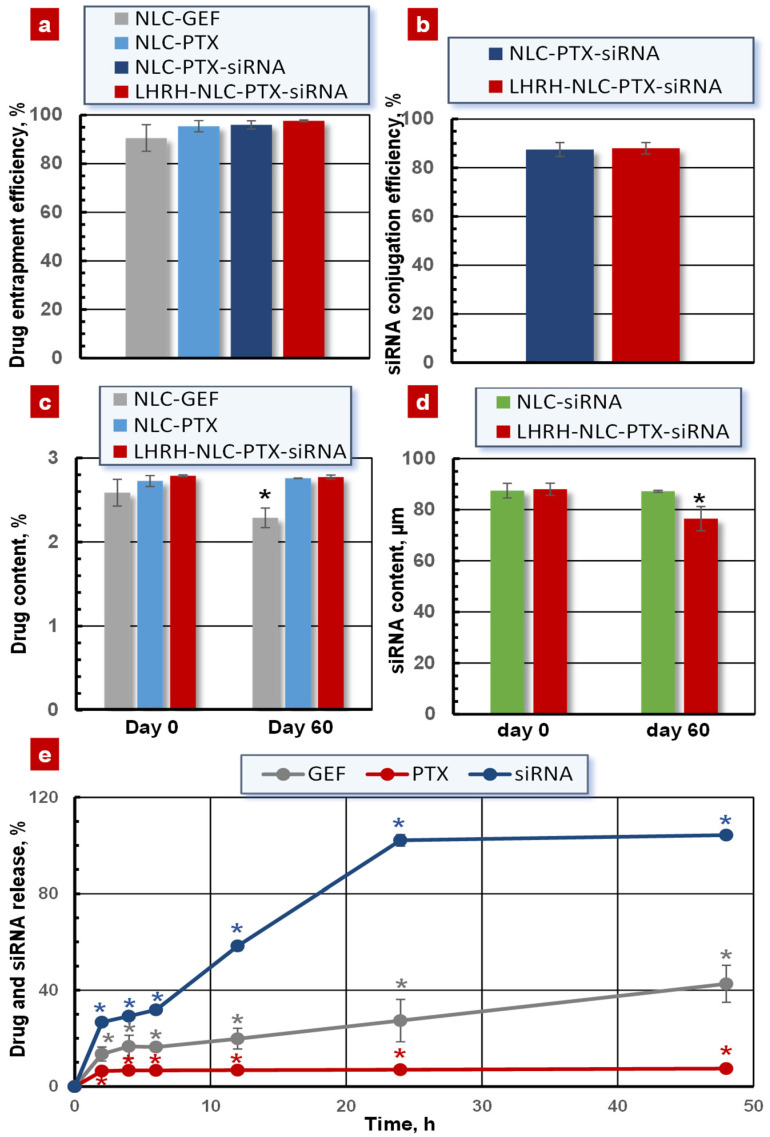
Drug/siRNA loading and release for different nanoparticles. (**a**) Entrapment efficiency of GEF and PTX; (**b**) Conjugation efficiency of siRNA; (**c**) Drug content during the storage in aqueous solution under physiological pH at 4 °C; (**d**) siRNA content during the storage in aqueous solution under physiological pH at 4 °C; (**e**) Drug release in aqueous solution under physiological pH and 37 °C. Means ± SD are shown. * *p* < 0.05 when compared with day 0.

As shown in Figure 6a, the entrapment efficiency of drugs gefitinib and paclitaxel was 90.54 ± 5.48% and 97.60 ± 0.34% for the NLC–GEF and LHRH–NLC–PTX–siRNA, respectively.

The conjugation efficiency of siRNA on the surface of the NLCs was measured using the SYBR Gold assay. The conjugation efficiency of siRNA was 89.30 ± 0.22% and 88.0 ± 2.60% for NLC–PTX–siRNA and LHRH–NLC–PTX–siRNA (Figure 6b). We also determined the drug-loading capacity of the NLCs. The loading capacity of gefitinib and paclitaxel was 2.58 ± 0.15% and 2.78 ± 0.01% for the NLC–GEF and LHRH–NLC–PTX–siRNA, respectively (Figure 6c). Both the drug and siRNA loading capacity into the NLCs only slightly changed after 60 days of storage at 4 °C (Figure 6c,d).

The in vitro release profiles of GEF, siRNA, and PTX from the NLCs were studied by the dialysis bag method [75]. The cumulative release rate of PTX from LHRH–NLC–PTX–siRNA was substantially slower when compared with NLC–GEF (Figure 6e). In contrast, almost all conjugated siRNAs were released from the LHRH–NLC–PTX–siRNA complex within 25–50 h of incubation of the NLC-based system in the aqueous solution under 7.4 pH and 37 °C.

### 3.5. In Vitro Cellular Uptake

**Figure 7:**
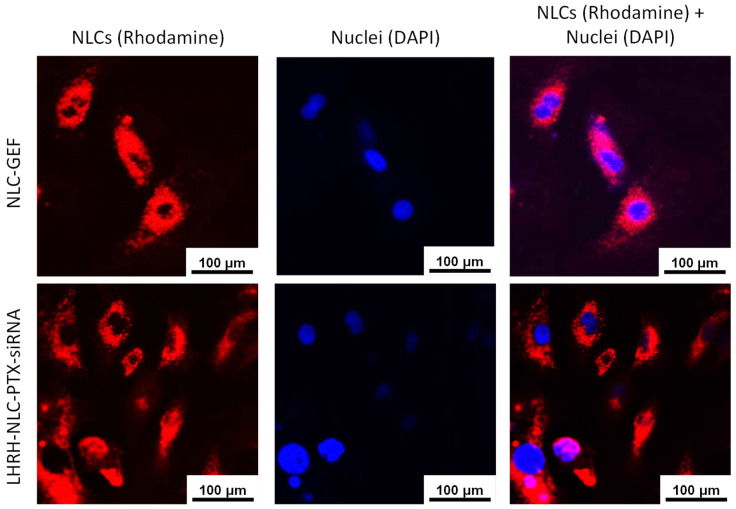
Representative fluorescence images of human NSCLC A549 cells incubated with rhodamine-labeled NLCs containing drugs and siRNA.

**Figure 8:**
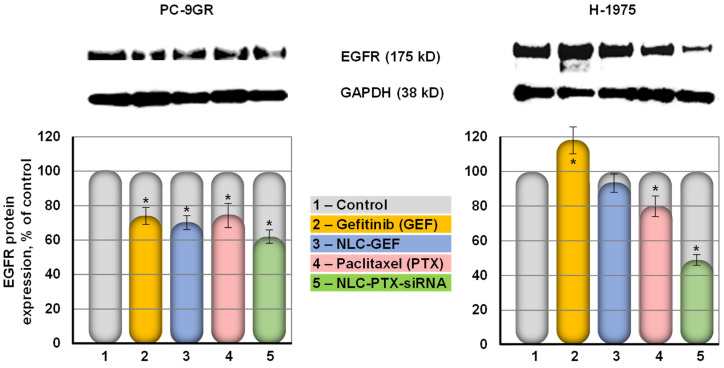
Expression of EGFR protein in human PC9-GR and H-1975 NSCLC cells treated with substances indicated. The expression of EGFR in the control non-treated cells was set to 100%. Top panel shows representative images of Western blots. GAPDH protein was used as internal standard. Means ± SD are shown. * *p* < 0.05 when compared with untreated control.

**Figure 9:**
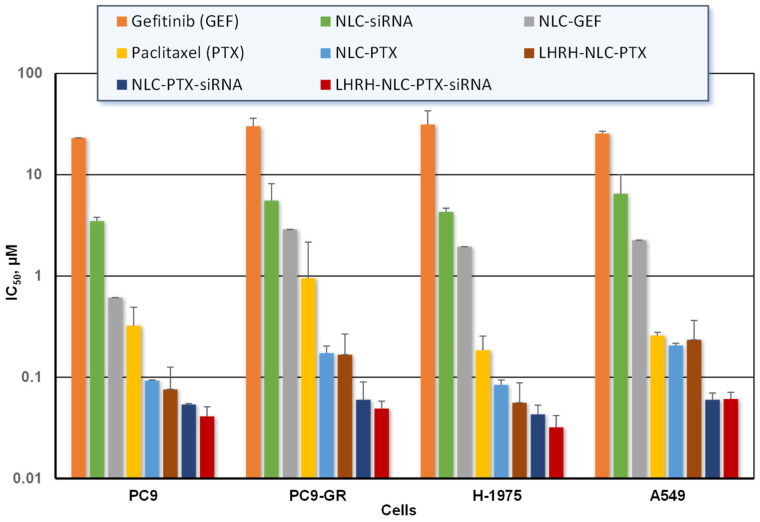
Cytotoxicity of free and NLC-bound drug formulations against various NSCLC cells. Means ± SD are shown.

In order to visualize NLCs in the cells, we labeled NLC by fluorophore rhodamine. A549 cells were incubated with rhodamine-labeled NLC formulations for 24 h and stained with DAPI for nuclei visualization. The data showed that the rhodamine-labeled NLCs containing anticancer drug and siRNA successfully penetrated into the cells and localized in the cytoplasm—indicating cellular uptake of nanoparticles (Figure 7). Our previous investigations showed that siRNA delivered by the similar NLCs were efficiently taken by cancer cells [23]. Our current data show that the delivered siRNA reduced the expression of targeted mRNA (Figure 8) and enhanced the toxicity of the entire NLC complex (Figure 9). These findings confirm the efficient cellular internalization of siRNA after the delivery by the developed NLCs and the preservation of siRNA-specific activity during its conjugation with NLCs, the delivery, and internalization by cancer cells.

### 3.6. Suppression of EGFR Protein in Lung Cancer Cells

Protein expression was studied using Western blot analysis of lysate samples of PC-9GR cells and H-1975 cells to probe the effect of NLC drug formulations on the expression of EGFR protein. PC-9GR cells possess intrinsic resistance to gefitinib, while H-1975 cells harbored the L858R and T790M mutations in the EGFR kinase domain [76]. We found that naked non-bound siRNA was practically not toxic for all studied lung cancer cells (IC_50_ dose could not be measured for all available concentrations of free siRNA). Moreover, in our previous experiments, we showed that naked siRNA did not penetrate cancer cells and did not influence the expression of a targeted gene [77]. Therefore, we did not include naked siRNA in our Western blotting experiments. Our data show that both non-bounded free and delivered with NLCs GEF decreased the expression of EGFR protein on about 20% in gefitinib-resistant PC-9GR cells (Figure 8). In contrast, free GEF was completely ineffective in human NSCLC H-1975 that express mutated EGFR protein. Moreover, the expression of EGF receptors increased in around 20% after the treatment with free GEF. The delivery of GEF by NLCs prevented such overexpression. The simultaneous delivery of PTX and siRNA targeted to EGFR mRNA further suppressed the expression of EGFR protein up to 40–50% in both types of NSCLC cells (Figure 8).

### 3.7. In Vitro Anticancer Efficiency of the NLC Drug Formulations

The anticancer efficiency of all studied NLC formulations were assessed by a MTT cell viability assay against various non-small cell human lung cancer A549, H-1975, PC-9, and PC-9GR cells. The percentage of live cells in a drug/NLC-treated sample was calculated by considering the absorbance of the vehicle-treated sample as 100%. Cytotoxicity (IC_50_ values) of GEF, PTX, and their NLC formulations in different NSCLC cells were summarized in Figure 9. GEF and PTX delivered by nanoparticles killed cancer cells much more effectively when compared with free non-bound drugs. Paclitaxel-loaded NLCs more effectively killed all studied cancer cells as compared to that of the gefitinib-loaded NLCs. The highest anticancer efficacy was found in all cells treated with the NLC–GEF and LHRH–NLC–PTX–siRNA (5 to 10-fold).

## 4. Discussion

In the present investigation, we developed a cancer-targeted multicomponent and multifunctional drug delivery system for the treatment of NSCLC, which expresses EGF receptors. The system combines several innovative approaches developed for treating lung cancer cells that overexpressed EGF receptors: (1) cancer targeting by a ligand (LHRH) to receptors overexpressed in cancer cells, which initiates receptor-mediated endocytosis and enhances the internalization of an entire system specifically by NSCLC while limiting adverse side effects upon healthy cells, tissues, and organs; (2) detecting cancer cells by an imaging agent (e.g., rhodamine for optical imaging); (3) suppression of EGF tyrosine kinase signaling pathways for existing EGF receptors by small molecule inhibitor(s) (e.g., gefitinib) in order to limit the proliferation, invasion, angiogenesis, and metastasis of cancer cells; (4) preventing the de novo synthesis of the EGFR protein through destroying its mRNA by the delivered siRNA; (5) induction of cell death by the incorporated lipophilic anticancer drug (paclitaxel). This work represents a direct extension of our previous investigations that clearly showed that the use of nanocarriers for the delivery of different drugs, antisense nucleotides, and siRNA significantly enhanced their anticancer activity [21,23,24,40]. The incorporation of anticancer drugs into nanocarriers increases drug stability and prevents its degradation during the journey to the site of action. The simultaneous inducing of cell death and inhibition of cellular defensive mechanisms and proliferation of cancer cells (e.g., by LHRH peptide) led to the dramatic increase the efficacy of anticancer drugs. The targeting of a highly toxic multifunctional delivery systems specifically to cancer cells further enhanced their cellular internalization and limits adverse side effects on healthy cells [21,55,58,59,61]. Moreover, such targeting reduces differences between various nanoparticles, allowing selecting a nanoparticle type and other features solely based on the characteristics of delivered active components, carrier stability, etc. [57]. In the present investigation, we selected nanostructured lipid carriers that possess an extended loading capacity for hydrophobic drugs (e.g., PTX). In order to further enhance the stability of NLCs, we used α-tocopherol as a liquid lipid phase of the NLC formulation. Tocopherol acts as an antioxidant in the lipid phase of cell membranes and improves the stability of NLCs in aqueous medium [78,79]. In addition to the high loading capacity for lipophilic compounds, NLCs as well as other lipid-based carriers (e.g., liposomes) are the most suitable for inhalation local delivery to lungs, which is very important and promising for the treatment of lung diseases [21,22,23,40,41,42]. 

The lipid composition of prepared NLCs was very similar in all synthetized types of delivery systems. The differences were between cancer-targeted and non-targeted systems and between negatively and positively charged ones. In cancer-targeted systems, approximately one-half of the DSPE–PEG surfactant was substituted for DSPE–PEG–LHRH, providing LHRH as a targeting moiety/ligand to LHRH receptors overexpressed in cancer cells and almost not expressed in normal cells in visceral organs [55,58]. This creates prerequisites for the specific delivery of cancer-targeted NLCs predominately to cancer cells and limiting adverse side effects upon healthy cells, tissues, and organs [21,58]. On the other hand, positively charged NLCs additionally contained around 5% of DOTAP for creating cationic NLCs, allowing a strong conjugation with anionic (at physiological pH) siRNA. It should be stressed that such a conjugation dramatically decreased the total positive charge of NLC–siRNA complexes for the safe +10–20 mV that does not induce negative effects on normal cells and tissues [80]. 

As expected, synthesized NLCs possessed high loading capacity for anticancer drugs and conjugation efficacy for siRNA. The former on average varied from 90 to 98% while the latter was close to 85–90%, making the proposed cancer-targeted NLCs comparable and even more advanced when compared with other lipid-based and other types of nanocarriers [35,36,39]. It should also be stressed that the proposed NLC formulations demonstrated a very low degradation and deprivation of drugs, especially PTX, during short- and long-term storage in aqueous solution with different ranges of pH, temperature, and freezing–thawing conditions. The nanoparticles also preserved their size, monodisperse distribution, and the total zeta potential during the storage. We have evaluated the stability of our lipid nanoparticles in various settings to see whether these nanoparticles could be useful for a wide range of conditions such as normal pH, gastric pH, long-time, and accelerated storage after three freeze–thaw cycles. It is well known (and our previous experimental data support this fact) that naked siRNA is very unstable even under the physiological conditions [20,77]. Therefore, the stability of an entire NLC formulation is very important in order to prevent the leakage of nucleic acid out from the nanoparticles and its degradation. The stability of encapsulated siRNA was estimated by two series of experiments. First, we measured the siRNA content inside the nanoparticles after 60 days of storage under physiological pH at 4 °C. It was found that only 10–15% of encapsulated siRNA was leaked out from the nanoparticles after two-month storage (Figure 6d). Second, we estimated the “quality” of the encapsulated siRNA by measuring its ability to suppress the targeted mRNA (Figure 8). We did not find significant differences in this suppression before and after the storage. These data allow us to conclude that the conjugation of siRNA with the nanoparticles dramatically increased its stability.

However, despite the unique aqueous stability, the cancer-targeted NLCs easily released their payload (drugs and siRNA) into the cellular cytoplasm after internalization by cancer cells via receptor-mediated endocytosis, when the plasma membrane forms a coated pit with the system inside, which in turn converts to endosomes and fuses with lysosomes, leading to the destruction of the NLC-based system [81]. It is generally believed that the so-called “proton sponge effect” plays a substantial role in the endosomal escape of siRNA from charged nanocarriers inside cells after endocytosis. Internalized NLCs–siRNA complexes (as well as any other polyplex) possess the proton-buffering capacity that triggers osmotically induced swelling of the endosome and rupturing the endosomal membrane and DOTAP–siRNA complexes, allowing the entry of free siRNA into the cytoplasm of cancer cells [82,83,84].

The data obtained allow for a comparison of two different strategies of suppression of EGFR-mediated signaling pathways: inhibition of receptor TK with NLC delivered small molecule drug GEF and the direct limitation of the expression of EGF receptors by siRNA. The results clearly demonstrate a substantial advantage of the latter method and show that siRNA targeted to EGFR and delivered by cancer-targeted NLCs led to a more pronounced suppression of these pathways and substantially higher cytotoxicity. This approach also significantly enhanced a cell death induction efficiency of anticancer drug PTX by the suppression of cellular defensive mechanisms associated with EGFR-mediated signaling pathways. Moreover, a positive effect of delivered siRNA does not depend on the presence or absence mutations in EGF receptors.

In addition, we found that paclitaxel statistically significantly decreased the expression of EGF receptors in different lung cancer cells. Although paclitaxel can suppress the EGFR signaling pathways in different cancer cells most probably via matrix metalloproteinases [85,86,87,88], a direct suppression of EGF receptors, in our knowledge, was not previously reported. In our studies, paclitaxel treatment reduced only about 10–15% expression of the EGFR protein when compared to the vehicle-treated cells. This could be attributed at least in part by a mediation of EGFR signaling in lung cancer cells and/or the inhibition of cellular metabolism by the drug. The detailed mechanisms of such phenomenon require additional investigations.

In these experiments, we did not set a task to show a role of a targeting moiety in increasing the anticancer effect of drug/siRNA-loaded nanoparticles. One could not expect a dramatic difference between cancer-targeted and non-targeted drug/siRNA-containing nanoparticles in the solution in vitro. In fact, the cancer cell targeting is designed to be used in vivo to increase drug/siRNA accumulation in tumor and limit the exposure of non-cancerous healthy cells. Previously, when we suggested the use of LHRH peptide for the first time as a cancer-targeting moiety, we carried out an extensive set of experiments to prove that such cancer targeting significantly enhanced antitumor activity and limited the severe adverse side effects on healthy organs, tissues, and cells in vivo [55,57,58,59].

Finally, it should be stressed that we did find an additive effect of codelivery of an anticancer drug (paclitaxel) and an inhibitor of EGF receptors in lung cancer cells. The results show that the combination of paclitaxel with siRNA targeted to EGF receptors enhanced the anticancer efficacy of the paclitaxel that cannot be achieved by the separate delivery of each component. These data provide the proof of concept of the proposed approach and clearly showed the advantage of a complex delivery system that simultaneously induces cell death by an anticancer drug and suppresses EFGR-mediated cellular defense in lung cancer cells. It should be stressed again that the anticancer effect of such a combination exceeds the efficacy of a traditional small molecule inhibitor of EGFR and, in contrast to gefitinib, its anticancer effect does not depend on the existence or absence of specific mutations of EGF receptors.

## 5. Conclusions

In conclusion, we report stable and multifunctional nanostructured lipid carriers (NLCs) comprised of therapeutic agents (anticancer drugs and human EGFR siRNA), imaging agents (fluorophore), and targeting agents (LHRH peptide) for the delivery of therapeutics specifically to human lung cancer cells. All the NLCs displayed a narrow size range with a single peak on the histogram, indicating a uniform distribution of the nanoparticles in the colloidal formulations. NLCs were found to be stable at storage conditions in aqueous buffer, cell culture media, as well as in low pH condition with almost no changes in particle size and the physical appearance. The drug entrapment efficiency (EE) of gefitinib and paclitaxel was greater than 90% (90.54 ± 5.48% and 97.60 ± 0.34%, respectively). The drug entrapment efficiency of these NLCs was almost intact even after 60 days of storage. Both the gefitinib and paclitaxel-loaded NLCs showed 5 to 10-fold improved in vitro anticancer activity in a series of human lung cancer A549, PC-9, PC-9GR, and H-1975 cells when compared with their parent drugs. Paclitaxel and the human EGFR siRNA-encapsulated LHRH–NLC–PTX–siRNA system reduced the expression of EGFR protein in cells with and without mutations of EGFR. Consequently, such a complex multifunctional delivery system can potentially represent an innovative theranostic strategy for the detection and treatment of drug-resistant NSCLC.

## Figures and Tables

**Table 1:** The list of all NLC formulations.

NLC Type	NLC Name
Empty NLC	NLC
Drug-loaded NLCs	NLC–GEF
	NLC–PTX
siRNA-loaded NLC	NLC–siRNA
Drug and siRNA-loaded NLC	NLC–PTX–siRNA
Drug-loaded and LHRH-targeted NLC	LHRH–NLC–PTX
Drug and siRNA-loaded and LHRH-targeted NLC	LHRH–NLC–PTX–siRNA

## Data Availability

Data presented in this study are available on request from the corresponding author.

## Supplementary Materials

The following are available online at https://www.mdpi.com/article/10.3390/pharmaceutics13071063/s1: Figure S1: ^1^H-NMR spectrum of the targeting ligand DSPE–PEG–LHRH peptide; Figure S2: Size distribution profile of the NLC formulations in aqueous phase; Figure S3: HPLC traces (retention time in minutes) of gefitinib (GEF) and paclitaxel (PTX) in standard sample and in the corresponding NLC formulations; Table S1: Stability of the NLC formulations under storage in RPMI-1640 culture media.
