# Supplementary material for: Multifunctional Lipid-Based Nanoparticles for Codelivery of Anticancer Drugs and siRNA for Treatment of Non-Small Cell Lung Cancer with Different Level of Resistance and EGFR Mutations
Source: Pharmaceutics. 2021 Jul 11;13(7):1063. doi: 10.3390/pharmaceutics13071063 (PMC8309189; doi:10.3390/pharmaceutics13071063)
Supplement: Supplementary file 1 [file pharmaceutics-13-01063-s001.zip › pharmaceutics-1250379-supplementary.pdf]

pharmaceutics

MDPI

Supplementary Materials: Multifunctional Lipid-Based Nanoparticles for Codelivery of Anticancer Drugs and siRNA for Treatment of Non-Small Cell Lung Cancer with Different Level of Resistance and EGFR Mutations

Joydeb Majumder and Tamara Minko

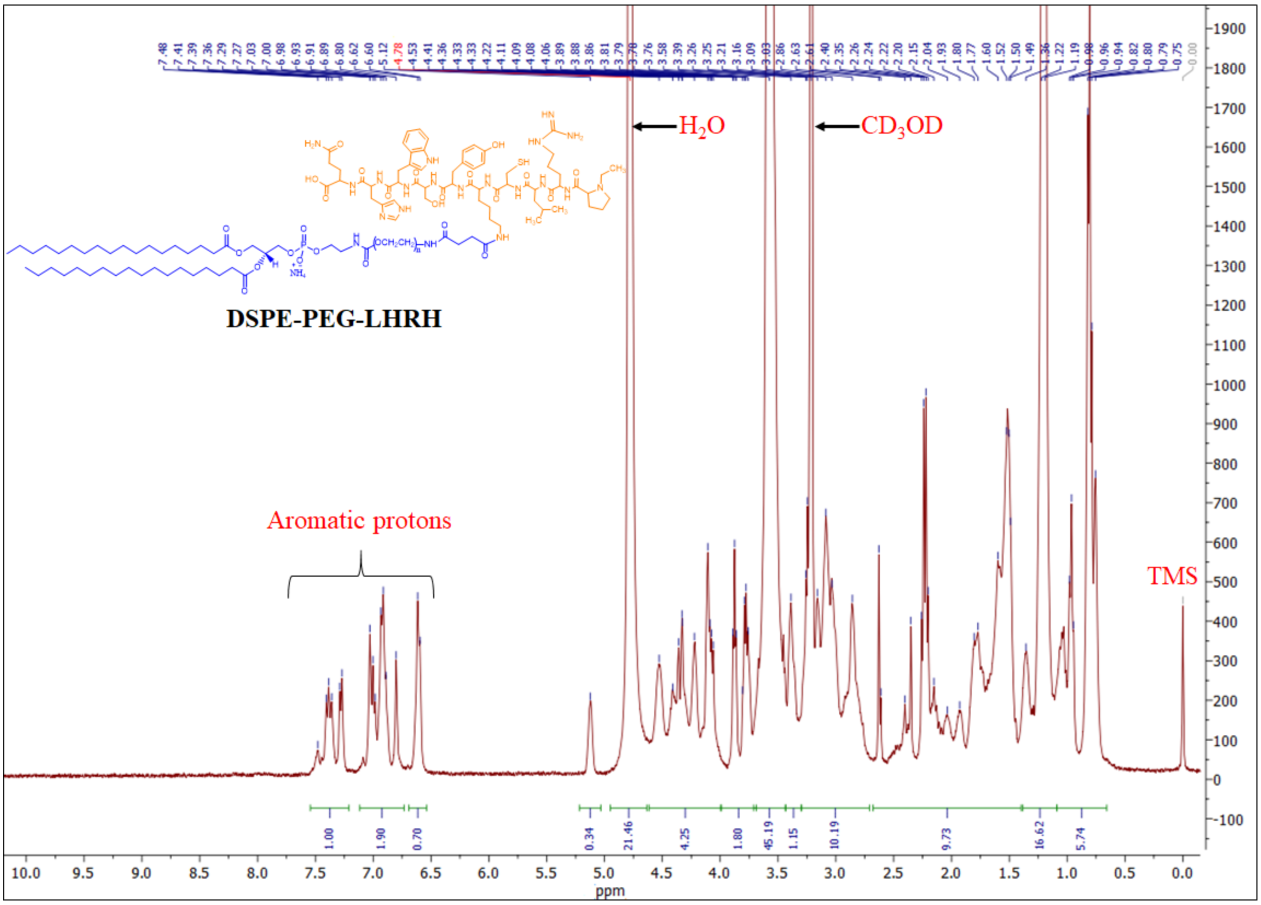

Figure S1. ^{1}H-NMR spectrum of the targeting ligand DSPE-PEG-LHRH peptide using CD_3OD solvent. 20 mg of DSPE-PEG-LHRH peptide was dissolved in 0.6 ml of deuterated methanol (CD_3OD). ^{1}HNMR data was recorded using 400 MHz Bruker NMR instrument at room temperature. Chemical shifts are shown in ppm. The reference standard tetramethyl silane (TMS) appeared at 0.0 ppm and the aromatic protons appeared at 6.5–7.5 ppm.

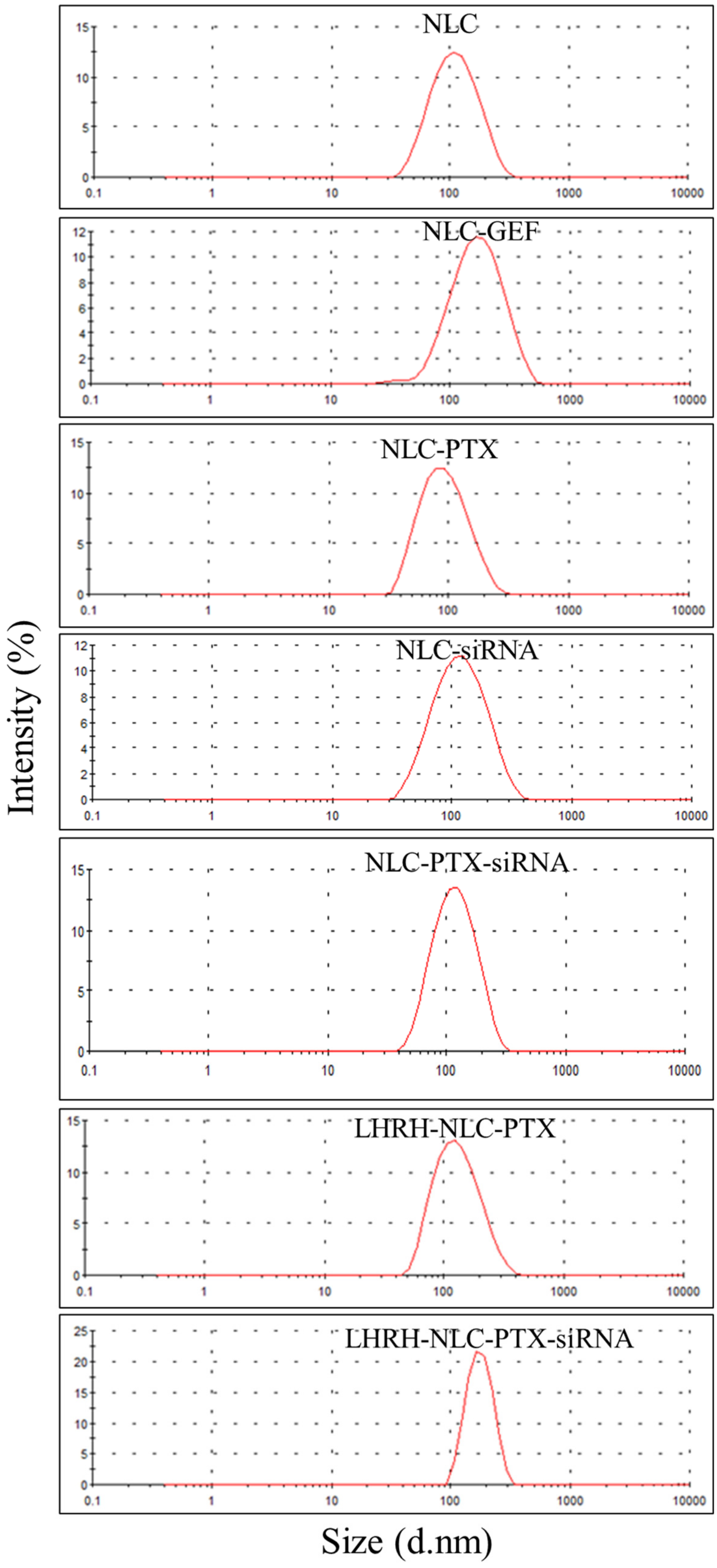

Figure S2. Size distribution profile of the NLC formulations in aqueous phase.

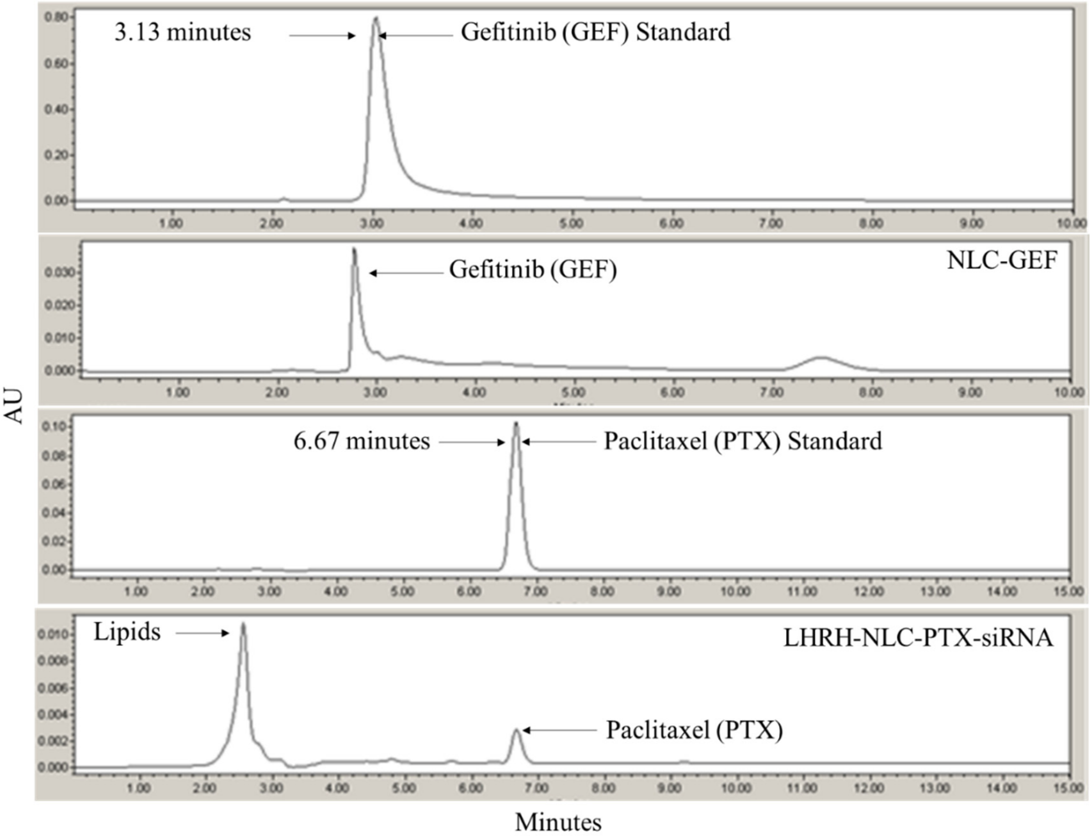

Figure S3. HPLC traces (retention time in minutes) of gefitinib (GEF) and paclitaxel (PTX) in standard sample and in the corresponding NLC formulations.

Table S1. Stability of the NLC formulations under storage in RPMI-1640 culture media.

Name of NLC	Time (Day)	Z-Average Size (nm)	PDI
NLC	Day 0	106.23 ± 1.07	0.242 ± 0.011
	Day 7	93.44 ± 0.80	0.226 ± 0.008
	Day 60	90.09 ± 1.22	0.221 ± 0.004
NLC-GEF	Day 0	149.00 ± 1.03	0.216 ± 0.019
	Day 7	137.96 ± 1.25	0.206 ± 0.004
	Day 60	136.80 ± 0.23	0.202 ± 0.005
NLC-PTX	Day 0	82.10 ± 0.52	0.157 ± 0.004
	Day 7	82.78 ± 0.12	0.169 ± 0.010
	Day 60	86.76 ± 1.41	0.195 ± 0.004
NLC-siRNA	Day 0	104.10 ± 2.23	0.158 ± 0.010
	Day 7	134.76 ± 4.16	0.362 ± 0.001
	Day 60	122.63 ± 0.57	0.270 ± 0.001
NLC-PTX-siRNA	Day 0	101.96 ± 0.51	0.158 ± 0.009
	Day 7	101.90 ± 0.61	0.169 ± 0.011
	Day 60	104.5 ± 0.49	0.180 ± 0.010
LHRH-NLC-PTX	Day 0	113.70 ± 0.69	0.148 ± 0.012
	Day 7	113.80 ± 0.69	0.150 ± 0.004
	Day 60	119.30 ± 1.31	0.184 ± 0.018
LHRH-NLC-PTX-siRNA	Day 0	153.13 ± 1.92	0.191 ± 0.008
	Day 7	161.86 ± 1.69	0.181 ± 0.006
	Day 60	159.26 ± 0.27	0.0246 ± 0.005
